# Five Years of Cenosumab Exposure in Women With Postmenopausal Osteoporosis: Results From the First Two Years of the FREEDOM Extension

**DOI:** 10.1002/jbmr.1479

**Published:** 2011-11-23

**Authors:** Socrates Papapoulos, Roland Chapurlat, Cesar Libanati, Maria Luisa Brandi, Jacques P Brown, Edward Czerwiński, Marc-Antoine Krieg, Zulema Man, Dan Mellström, Sebastião C Radominski, Jean-Yves Reginster, Heinrich Resch, José A Román Ivorra, Christian Roux, Eric Vittinghoff, Matthew Austin, Nadia Daizadeh, Michelle N Bradley, Andreas Grauer, Steven R Cummings, Henry G Bone

**Affiliations:** 1 Department of Endocrinology & Metabolic Diseases, Leiden University Medical CenterLeiden, The Netherlands; 2 INSERM UMR 1033, Université de LyonLyon, France; 3 Amgen Inc.Thousand Oaks, CA, USA; 4 Department of Internal Medicine, University of FlorenceFlorence, Italy; 5 Department of Medicine, Laval University and Rheumatology Division, CHUQ Research Centre, Laval UniversityQuebec City, QC, Canada; 6 Krakow Medical CenterKrakow, Poland; 7 Bone and Joint Department, University Hospital of LausanneLausanne, Switzerland; 8 Endocrinology Division, Centro TIEMPOBuenos Aires, Argentina; 9 Osteoporosis Clinic, Center for Bone Research, Sahlgrenska University HospitalGöteborg, Sweden; 10 Department of Internal Medicine, Universidade Federal do ParanáCuritiba, Brazil; 11 Bone and Cartilage Metabolism Unit, University of LiègeLiège, Belgium; 12 Medical Department II, St. Vincent HospitalVienna, Austria; 13 Rheumatology Unit, Hospital Universitario La FeValencia, Spain; 14 Department of Rheumatology, Cochin Hospital, Paris Descartes UniversityParis, France; 15 Department of Epidemiology and Biostatistics, University of California San FranciscoSan Francisco, CA, USA; 16 San Francisco Coordinating Center, CPMC Research Institute, and University of California San FranciscoSan Francisco, CA, USA; 17 Michigan Bone and Mineral ClinicDetroit, MI, USA

**Keywords:** BONE MINERAL DENSITY, BONE TURNOVER MARKERS, DENOSUMAB, FRACTURE, PIVOTAL FRACTURE TRIAL EXTENSION

## Abstract

The 3-year FREEDOM trial assessed the efficacy and safety of 60 mg denosumab every 6 months for the treatment of postmenopausal women with osteoporosis. Participants who completed the FREEDOM trial were eligible to enter an extension to continue the evaluation of denosumab efficacy and safety for up to 10 years. For the extension results presented here, women from the FREEDOM denosumab group had 2 more years of denosumab treatment (long-term group) and those from the FREEDOM placebo group had 2 years of denosumab exposure (cross-over group). We report results for bone turnover markers (BTMs), bone mineral density (BMD), fracture rates, and safety. A total of 4550 women enrolled in the extension (2343 long-term; 2207 cross-over). Reductions in BTMs were maintained (long-term group) or occurred rapidly (cross-over group) following denosumab administration. In the long-term group, lumbar spine and total hip BMD increased further, resulting in 5-year gains of 13.7% and 7.0%, respectively. In the cross-over group, BMD increased at the lumbar spine (7.7%) and total hip (4.0%) during the 2-year denosumab treatment. Yearly fracture incidences for both groups were below rates observed in the FREEDOM placebo group and below rates projected for a “virtual untreated twin” cohort. Adverse events did not increase with long-term denosumab administration. Two adverse events in the cross-over group were adjudicated as consistent with osteonecrosis of the jaw. Five-year denosumab treatment of women with postmenopausal osteoporosis maintained BTM reduction and increased BMD, and was associated with low fracture rates and a favorable risk/benefit profile. © 2012 American Society for Bone and Mineral Research

## Introduction

Increased bone resorption after menopause leads to loss of bone mass and microstructural deterioration which significantly increase fracture risk. Receptor activator of NF-κB (RANK) ligand plays an essential role in mediating resorption through osteoclast formation, function, and survival.(1, 2) Denosumab is a fully human monoclonal antibody that inhibits RANK ligand.

In the pivotal, 3-year, placebo-controlled FREEDOM trial,(3) administration of 60 mg denosumab subcutaneously every 6 months to postmenopausal women with osteoporosis significantly reduced bone turnover markers (BTMs), increased bone mineral density (BMD), and reduced new vertebral, hip, and nonvertebral fractures by 68%, 40%, and 20%, respectively.

Osteoporosis is a chronic disease requiring long-term treatment. Therefore, characterization of the long-term efficacy and safety of denosumab is essential for clinical practice. Accordingly, FREEDOM has been extended, in an open-label design, for an additional 7 years, during which all participants receive denosumab. The extension includes two populations: those who had received denosumab for 3 years during the core trial (long-term group) and those who had received placebo for 3 years during the core trial (cross-over group). We report the effects of denosumab on BTMs, BMD, fracture rates, and safety for the first 2 years of the extension.

## Patients and Methods

### Study design

**Fig. 1:**
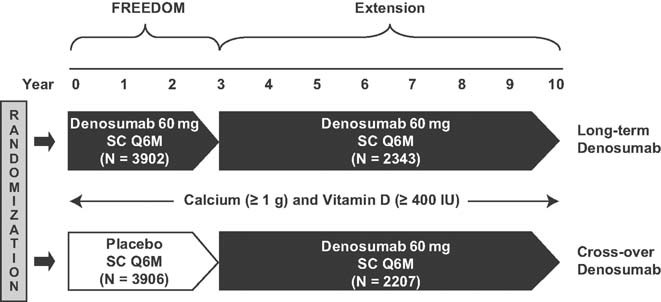
Study design showing the 3-year FREEDOM study and the 7-year extension. The data reported here are for the first 2 years of the extension study. SC = subcutaneous; Q6M = every 6 months.

The FREEDOM core trial design and results have been published.(3) In short, FREEDOM was a phase 3, randomized, double-blind, placebo-controlled, 3-year, global study. Enrolled women were aged 60 to 90 years with a lumbar spine or total hip BMD T-score between −2.5 and −4.0 at either site. Participants were randomized to receive 60 mg denosumab (Prolia®; Amgen Inc., Thousand Oaks, CA) or placebo subcutaneously every 6 months for 3 years, in addition to daily calcium (≥1 g) and vitamin D (≥400 IU). All women who completed the core trial (ie, completed their 3-year visit, did not discontinue investigational product, and did not miss >1 dose) were eligible to enter the extension. During the extension, all participants are scheduled to receive 60 mg denosumab subcutaneously every 6 months for 7 years with daily calcium and vitamin D (Fig. 1). The study is ongoing and the preplanned data analyses reported here include the first 2 years of the extension, representing up to 5 years of continued denosumab exposure.

The study protocol was approved by an ethics committee or institutional review board for each site. Participants provided written informed consent. Representatives of the sponsor, Amgen Inc., designed the study in collaboration with investigators, and conducted the statistical analysis according to a prespecified plan.

### Study procedures

In the 7-year extension trial, main study visits are scheduled to occur at baseline (corresponding to the end of the core trial) and every 6 months for 7 years. Participants are scheduled to receive 60 mg denosumab subcutaneously every 6 months (±1 month) for 7 years beginning on day 1. For this report, all efficacy and safety assessments continued with the same methodology used during FREEDOM.(3) Measurements of serum C-terminal telopeptide of type 1 collagen (CTX) and procollagen type I N-terminal propeptide (P1NP) were obtained from a subset of women who participated in the FREEDOM BTM substudy and continued in the extension (CTX: baseline, day 10, and months 3, 4, 6, 12, and 24; P1NP: baseline, day 10, and months 6, 12, and 24). Undetectable values were imputed using the corresponding assay's established lower limit of detection value (CTX, 0.049 ng/mL; P1NP, 10 µg/L) as previously reported.(4) BMD measurements were performed by dual-energy X-ray absorptiometry (DXA) at the lumbar spine and proximal femur (all women) and at the 1/3 radius (subset of women). Vertebral fractures were identified by a central facility (Synarc, Inc.) using the Genant semiquantitative grading scale using thoracic and lumbar lateral radiographs obtained at extension baseline and year 2.(5) A prevalent fracture at baseline was defined as a vertebral body with a semiquantitative grade of ≥1. As was prespecified in the original protocol, a new vertebral fracture was identified when there was ≥1 grade increase from a previous grade of 0 in any vertebra between T4 and L4, excluding fractures associated with high trauma severity or a pathologic fracture. Nonvertebral fractures included low-trauma fragility fractures only, as defined previously.(3) Clinical fractures required confirmation by diagnostic imaging or a radiologist's report. At all study visits, adverse events (AEs), clinical fracture information, and concomitant medications were recorded. Each potential case of osteonecrosis of the jaw (ONJ) was reviewed by an independent, masked, external, expert adjudication committee, as described previously.(6)

### Statistical analyses

The primary objective of the extension is to describe the safety and tolerability of denosumab. Secondary objectives include evaluation of the changes in BTMs and BMD, and the incidence of vertebral and nonvertebral fractures.

Safety analyses included participants who received ≥1 dose of investigational product. New adverse events occurring during the extension were coded using the Medical Dictionary for Regulatory Authorities (MedDRA v13.0). For consistency, MedDRA v13.0 will be used for coding and reporting adverse events for the duration of the extension study. The analyses of AEs were descriptive and included exposure-adjusted subject incidence rates. Analyses of BTM percent change from baseline required observed values at baseline and the time points of interest. Results are presented as medians and interquartile ranges. Analyses of BMD percent change from baseline required observed values at baseline and the time points of interest. Percent changes in BMD were analyzed using the likelihood-based repeated measures model(7) including treatment, age stratification variable, visit, baseline value, densitometer type, treatment-by-visit interaction, and baseline value-by-densitometer type interaction as fixed effects using an unstructured variance-covariance structure. Visit was treated as a categorical variable. Kenward and Roger's approach(8) was followed for estimating the denominator degrees of freedom for the hypothesis test. Results are presented as least squares means with 95% confidence intervals. Finally, we calculated crude subject incidence rates for new vertebral fracture (multiple fracture occurrences were counted once per subject) and Kaplan-Meier estimates of the cumulative incidence of nonvertebral fracture.

Because all subjects in the extension received denosumab, we used a simulation method developed for such an extension study design(9) to estimate expected fracture rates in a hypothetical cohort of long-term placebo controls (so-called “virtual twins”), with the baseline characteristics of the long-term denosumab subjects. First, linear, Poisson, and logistic prediction models were developed using actual FREEDOM data on BMD, fracture history, body mass index, age, and smoking status for subjects who received placebo during the 3 years of FREEDOM and enrolled in the extension study. These models were then used to predict fracture outcomes for the denosumab-treated women who entered the extension had they received placebo for 5 years (virtual twins).

Final FREEDOM data were reported previously,(3) and are summarized in this manuscript to illustrate the extension results. For BTMs and BMD, only data from women enrolled in the extension are presented.

## Results

**Fig. 2:**
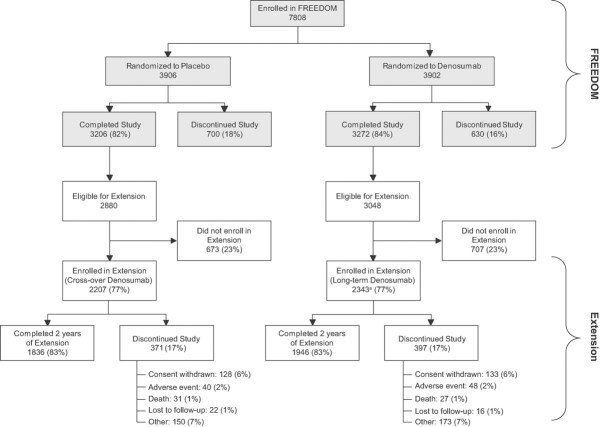
Disposition of all participants. All women who completed FREEDOM (ie, completed their 3-year visit, did not discontinue investigational product [IP], and did not miss >1 dose) were eligible to participate in the FREEDOM extension. ^a^Two women who discontinued denosumab also entered the extension in the long-term denosumab group.

Of the 7808 participants enrolled in the core trial, 5928 (76%) were eligible for enrollment in the extension (ie, completed their 3-year visit, did not discontinue investigational product [IP], and did not miss >1 dose) (Fig. 2). Of these, 4550 (2343 long-term, 2207 cross-over) enrolled in the extension, corresponding to 77% of those eligible to enroll and 58% of those enrolled in the original FREEDOM study. By year 2, 3782 (83% of those enrolled) remained on study. The long-term and cross-over groups address two distinct considerations: (1) the impact of continued denosumab administration past 3 years, and (2) the reproducibility of the FREEDOM results. Each group is described separately below.

### Long-term denosumab group

#### Baseline characteristics

FREEDOM and extension baseline characteristics for the participants in the long-term group are shown in Table 1. At the start of the extension, 53.7% were ≥75 years old. The proportion of patients with prevalent vertebral fractures was similar (23.9% at FREEDOM baseline versus 24.5% at extension baseline). At the extension baseline, the BMD T-scores were improved and the BTM values remained low.

**Table 1:** Baseline Characteristics

	Long-term denosumab treatment, Extension subjects (*N* = 2343)		Cross-over denosumab treatment, Extension subjects (*N* = 2207)	
		
	FREEDOM baseline	Extension baseline	FREEDOM baseline	Extension baseline
Age (years)	71.9 (5.0)	74.9 (5.0)	71.8 (5.1)	74.8 (5.1)
Age groups, *n* (%)				
≥65 years	2209 (94.3)	2294 (97.9)	2067 (93.7)	2149 (97.4)
≥75 years	662 (28.3)	1258 (53.7)	624 (28.3)	1151 (52.2)
Years since menopause	23.7 (7.3)	26.7 (7.3)	23.7 (7.4)	26.7 (7.4)
Prevalent vertebral fractures, *n* (%)	559 (23.9)	573 (24.5)	485 (22.0)	551 (25.0)
Lumbar spine BMD T-score	−2.83 (0.67)	−2.14 (0.80)	−2.84 (0.68)	−2.81 (0.75)
Total hip BMD T-score	−1.85 (0.79)	−1.50 (0.79)	−1.85 (0.79)	−1.93 (0.80)
CTX^a^ (ng/mL), median (IQR)	0.524 (0.363–0.710)	0.183 (0.081–0.556)	0.554 (0.420–0.657)	0.568 (0.413–0.718)
P1NP^a^ (µg/L), median (IQR)	46.7 (34.0–58.2)	17.5 (11.0–26.0)	54.2 (40.0–65.7)	48.8 (35.0–65.8)

Data are mean with standard deviations (SD) unless otherwise noted.

BMD = bone mineral density; CTX = C-terminal telopeptide of type 1 collagen; IQR = interquartile range; *N* = number of subjects enrolled in the extension; P1NP = procollagen type I N-terminal propeptide.

a BTM subsets include 65 subjects in the long-term group and 36 subjects in the cross-over group.

#### BTMs

**Fig. 3:**
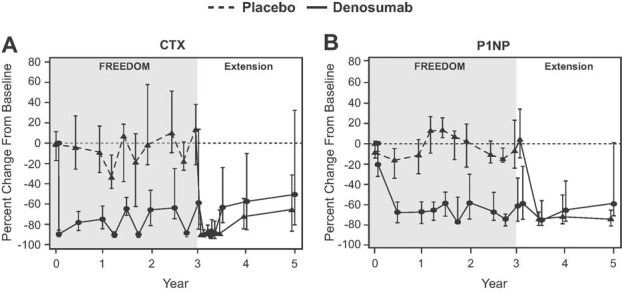
Percent change in bone turnover markers during FREEDOM and the extension. Changes in serum C-terminal telopeptide of type 1 collagen (CTX; panel *A*) and serum procollagen type I N-terminal propeptide (P1NP; panel *B*) are shown for 101 subjects (36 cross-over, 65 long-term) who were included in a substudy of bone turnover markers. Data are median (interquartile range).

Changes in serum CTX and P1NP are shown for 65 long-term denosumab women who participated in the BTM substudy (Fig. 3). After the seventh dose of denosumab (the first extension dose), reductions in CTX during the first 4 months and P1NP at 6 months were consistent with those observed after the first dose of denosumab during the core trial. The mean and median BTM values remained below FREEDOM pretreatment values throughout the 5 years of continued denosumab treatment. The reduction of BTMs showed an attenuation at the end of the dosing interval, as expected based on denosumab pharmacokinetics.(4) Although the small number of subjects with BTM values limits the robustness of the interpretation, the increases in BTMs at the end of the dosing interval appeared to increase with time in the study, an observation also reported within FREEDOM.(4)

#### BMD

**Fig. 4:**
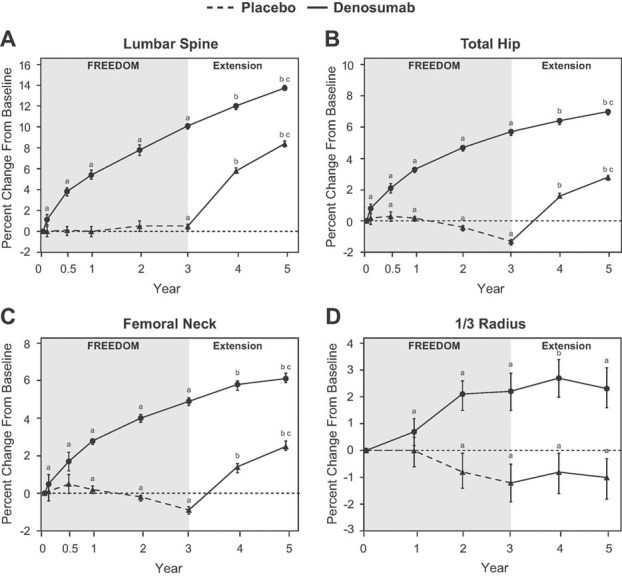
Percent change in bone mineral density (BMD) during FREEDOM and the extension. Changes in BMD at the lumbar spine (*A*), total hip (*B*), femoral neck (*C*), and 1/3 radius (*D*) are shown. Data are least squares means (95% CI). ^a^*p* < 0.05 compared with FREEDOM baseline; ^b^*p* < 0.05 compared with FREEDOM baseline and extension baseline. ^c^*p* < 0.05 compared with year 4.

Despite the apparent increase over time in the release of BTMs, there were further significant gains in BMD during the fourth and fifth years of denosumab treatment at the lumbar spine (1.9% and 1.6%, respectively), total hip (0.8% and 0.6%, respectively), and femoral neck (0.9% and 0.4%, respectively) (Fig. 4). A significant gain in BMD at the 1/3 radius was also observed during the fourth year of treatment (0.6%) but not during the fifth year (−0.3%). Increases in BMD over 5 years of continued denosumab treatment reached 13.7% (lumbar spine), 7.0% (total hip), 6.1% (femoral neck), and 2.3% (1/3 radius) (all *p* < 0.0001).

#### Fractures

**Fig. 5:**
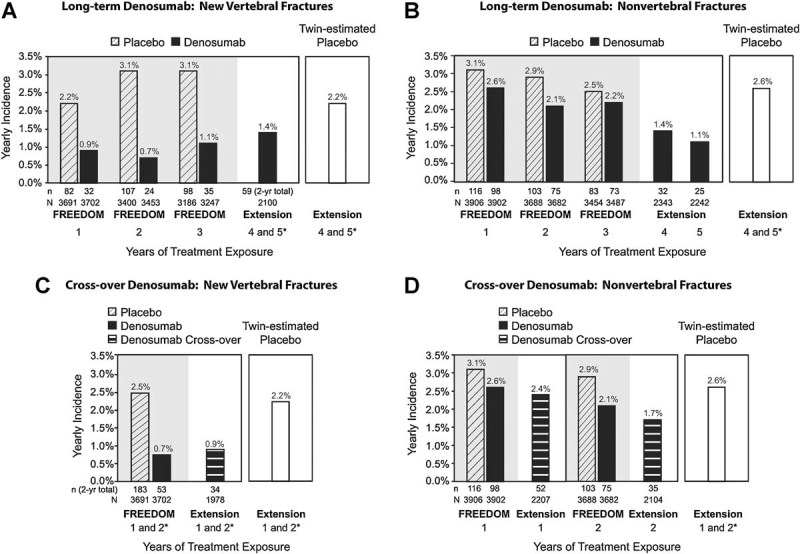
Yearly incidence of new vertebral fractures (*A* and *C*) and nonvertebral fractures (*B* and *D*) during FREEDOM and the extension. *n* = number of subjects with ≥1 fracture. *N* = number of subjects in the primary efficacy analysis set who were still on study at the beginning of each period. *Annualized rate: (2-year rate/2).

During FREEDOM, denosumab reduced the risk of new vertebral and nonvertebral fractures during each year of the trial, compared with placebo (Fig. 5A, B). In the extension, fracture incidence rates remained low and below those observed in the core trial placebo group. They also were below the estimated fracture incidence rates expected had the denosumab subjects who enrolled in the extension received placebo (twin-estimated placebo). Specifically, 2.8% (*n* = 59) of the women in the long-term denosumab group experienced ≥1 new vertebral fracture through year 2 of the extension (annualized rate of 1.4% for the fourth and fifth years of denosumab treatment). Fourteen women had a clinical vertebral fracture. Additionally, 1.4% and 1.1% of women in the long-term denosumab group experienced a nonvertebral fracture during the fourth and fifth years of denosumab exposure. The most common nonvertebral fractures in the long-term group during the first 2 years in the extension were wrist (*n* = 21), rib (*n* = 9), hip (*n* = 7), and ankle (*n* = 7) (with *n* = the number of affected women).

#### Adverse events

In the long-term group, the subject incidence rates per 100 subject-years for all, serious, and fatal AEs during the extension were similar to or lower than those in the placebo and denosumab groups during the core trial (Table 2). For example, the subject incidence rates of AEs reported by the placebo and denosumab groups during the core trial were 156.1 and 154.3, respectively, compared with 113.2 in the long-term denosumab group during the extension. The subject incidence rates of serious AEs were 10.4, 10.6, and 10.8 in the FREEDOM placebo, FREEDOM denosumab, and extension long-term denosumab groups, respectively. Rates of skin infection were low. There were no atypical femoral fractures or adjudicated ONJ events in the long-term group during the first 2 years of the extension.^1^

**Table 2:** Exposure-Adjusted Subject Incidence of Adverse Events

	Placebo	Denosumab		
		
	FREEDOM, years 1–3 (*N* = 3883), rate (n)	FREEDOM, years 1–3 (*N* = 3879), rate (n)	Extension, long-term, years 4–5 (*N* = 2343), rate (n)	Extension, cross-over, years 1–2 (*N* = 2206), rate (n)
Adverse events	156.1 (3614)	154.3 (3598)	113.2 (1955)	111.4 (1826)
Infection	30.7 (2113)	29.3 (2052)	25.1 (875)	27.4 (886)
Malignancy	1.6 (167)	1.8 (187)	2.0 (87)	1.6 (68)
Eczema	0.6 (67)	1.1 (119)	1.1 (47)	0.9 (39)
Hypocalcemia	<0.1 (3)	0.0 (0)	<0.1 (1)	0.1 (5)
Pancreatitis	<0.1 (3)	<0.1 (7)	<0.1 (1)	<0.1 (1)
Serious adverse events	10.4 (974)	10.6 (1002)	10.8 (442)	11.1 (428)
Infections	1.3 (134)	1.5 (160)	1.2 (55)	1.5 (63)
Cellulitis or erysipelas	<0.1 (1)	0.1 (12)	<0.1 (3)	<0.1 (1)
Fatal adverse events	0.8 (90)	0.6 (70)	0.6 (26)	0.8 (32)

Treatment groups are based on the original randomized treatments received in FREEDOM. All subjects in the extension are receiving denosumab.

*n* = total number of subjects with an adverse event; *N* = number of subjects who received ≥1 dose of investigational product; rate = exposure-adjusted subject incidence per 100 subject-years.

### Cross-over denosumab group

#### Baseline characteristics

Characteristics of the cross-over denosumab group at FREEDOM and extension baseline are shown in Table 1. At the start of the extension, 52.2% were ≥75 years old. Prevalent vertebral fracture rates in the placebo participants increased during the core trial as reflected in the baseline characteristics for this group: 25.0% at extension baseline versus 22.0% at FREEDOM baseline. The cross-over baseline BTM values and BMD T-scores were similar to core trial baseline values, consistent with the treatment of these participants with calcium and vitamin D during the core trial.

#### BTMs

Changes in serum CTX and P1NP are shown for 36 cross-over women who participated in the BTM substudy (Fig. 3). Following the initial administration of denosumab, a rapid and marked reduction in serum CTX occurred, followed by a reduction in serum P1NP. Both changes were nearly identical with those observed in the denosumab group at the beginning of the core trial. At the end of the dosing interval, attenuation of bone turnover reduction was observed, as seen during FREEDOM.(4) Throughout the first 2 years of denosumab treatment, the BTMs remained below their pretreatment values.

#### BMD

BMD increased significantly following denosumab administration (Fig. 4). BMD gains from extension baseline were 7.7% (lumbar spine), 4.0% (total hip), 3.3% (femoral neck), and 0.3% (1/3 radius) after 2 years of denosumab administration. Similar BMD gains were observed in the FREEDOM denosumab group during the first 2 years: 7.8% (lumbar spine), 4.7% (total hip), 4.0% (femoral neck), and 2.1% (1/3 radius).

#### Fractures

The yearly incidence (annualized rate) of new vertebral fractures for the cross-over group was 0.9% (*n* = 34), similar to the 0.7% observed in the core trial for the first 2 years for the group that received denosumab (Fig. 5*C*). Three women had a clinical vertebral fracture. For nonvertebral fractures, the yearly incidence for the cross-over group was 2.4% and 1.7% during years 1 and 2 of the extension, respectively. These rates also were similar to the nonvertebral fracture incidences in the FREEDOM denosumab group and were below the year 1 and 2 nonvertebral fracture rates observed in the FREEDOM placebo group (Fig. 5*D*). Both the new vertebral and nonvertebral fracture rates for the cross-over group were below the estimated fracture incidence for the virtual twin-estimated placebo group. The most common nonvertebral fractures in the cross-over group during the first 2 years in the extension were wrist (*n* = 30), hip (*n* = 14), ankle (*n* = 13), and foot (*n* = 9).

#### Adverse events

The subject incidences of all, serious, and fatal AEs in the cross-over group during the first 2 years of the extension were similar to or lower than those reported by the FREEDOM placebo and denosumab groups (Table 2). For example, the subject incidence rates of AEs reported by the placebo and denosumab groups during the core trial were 156.1 and 154.3, respectively, compared with 111.4 for the cross-over denosumab group during the extension. The subject incidence rates of serious AEs were 10.4, 10.6, and 11.1 for the FREEDOM placebo, FREEDOM denosumab, and extension cross-over denosumab groups, respectively. There were no atypical femoral fractures. Two oral AEs (bone necrosis and osteomyelitis) were adjudicated as consistent with ONJ. Both lesions healed completely: one within 137 days and the other within 227 days. In one woman, healing occurred within the 6-month dosing interval and she continued to receive denosumab (two further doses) without further oral events. In the other woman, healing occurred after the 6-month dosing interval and denosumab was permanently discontinued. There was only one serious skin infection (erysipelas) in the cross-over group.

## Discussion

The extension trial enrolled women who had received denosumab or placebo in the FREEDOM trial,(3) and therefore provides an opportunity to evaluate the long-term efficacy and safety of continued denosumab administration (long-term group), and to reproduce the denosumab data observed in FREEDOM during the first 2 years of therapy (cross-over group).

BMD and BTMs are the main intermediate efficacy measures that are associated with anti-fracture efficacy of antiresorptive drugs for the treatment of osteoporosis.(10) The present study demonstrates that in participants who continued denosumab for a fourth and fifth year, the bone density measurements continued to increase, and the turnover markers were maintained at substantially lower than pretreatment levels, but no lower than those achieved early in the core trial. The data from the cross-over group confirmed the findings from the FREEDOM trial.

Phase three trials for anti-osteoporotic agents generally have placebo control arms, but the placebo assignment cannot be continued indefinitely in consideration of the well-being of those participants.(11–13) However, long-term exposure is important for assessment of both safety and efficacy of this chronic condition. The efficacy assessments are limited by the lack of an ongoing control group. Projections based on the fracture rate of the placebo group, adjusted for age, have been used as a referent in an effort to address this problem.(14) Subsequently, a more sophisticated model has been developed and validated.(9) In the FREEDOM study, the use of the placebo group to generate data for such a model was planned, and for the present report, this independently-validated model was used to project fracture rates for a virtual group of placebo-treated “twins” of the participants. This model takes into account several risk factors. While this method provides a useful tool for assessing treatment efficacy, it rests on the unverifiable assumption that outcomes for the virtual twins can be predicted without bias from the available data. Simulations suggest that the method is reasonably robust against selection bias, but vulnerable to secular trends in the underlying fracture risk.(9) Although there are inherent limitations to the inferences drawn from such models, fracture rates for the extension and cross-over groups were consistently below these estimates.

A main objective of the extension is to describe the safety of denosumab. The subject incidences of all, serious, and fatal AEs during the first 2 years of the extension were similar to or lower than those reported by the FREEDOM placebo and denosumab groups. Incidences of malignancy and infection (such as cellulitis or erysipelas) were low and showed no trend toward increases over time, and there was only a single serious skin infection in the cross-over group. There were no atypical femoral fractures in either group. Although cases of ONJ have now been observed in patients receiving denosumab, the incidence remains low.

Thus, our findings demonstrate a progressive increase in bone density and sustained but not progressive decrease in bone turnover, and are consistent with maintenance of anti-fracture efficacy. There was no apparent increase in the risk of AEs with extended exposure. The cross-over group sustained a single serious skin infection and two events adjudicated as ONJ, while confirming the previous efficacy observations. Denosumab remained a useful medication for osteoporosis over 5 years of exposure.

## Disclosures

This study was funded by Amgen Inc. SP and HR have received consulting fees from Amgen Inc. RC, MLB, J-YR, CR, and SRC have received research grants and consulting fees from Amgen Inc. CL, MA, ND, MNB, and AG are Amgen Inc. employees and own Amgen Inc. stock and/or stock options. JPB has received research grants and consulting fees from Amgen Inc. and is a member of the speaker's bureau for Amgen Inc. EC has received research grants and speaking fees from Amgen Inc. M-AK, DM, JAR, and EV have no financial conflicts to disclose. ZM and SCR have received research grants from Amgen Inc. HGB has received research grants, consulting fees, and speaking fees from Amgen Inc.

## Footnotes

1 Prior to submission of the manuscript, two cases adjudicated as ONJ were observed in the long-term group in the sixth year of this planned 10-year study.
